# Impact analysis of keyword extraction using contextual word embedding

**DOI:** 10.7717/peerj-cs.967

**Published:** 2022-05-30

**Authors:** Muhammad Qasim Khan, Abdul Shahid, M. Irfan Uddin, Muhammad Roman, Abdullah Alharbi, Wael Alosaimi, Jameel Almalki, Saeed M. Alshahrani

**Affiliations:** 1 Institute of Computing, Kohat University of Science & Technology, Kohat, Kohat, Pakistan; 2 Department of Information Technology, College of Computers and Information Technology, Taif University, Taif, Saudi Arabia; 3 Department of Computer Science, College of Computer in Al-Leith, Umm Al-Qura University, Makkah, Saudi Arabia; 4 College of Computing and Information Technology, Shaqra University, Shaqra, Saudi Arabia

**Keywords:** Text Rank, Yake, TF-IDF, Keyword extraction, Contextual Word Embedding

## Abstract

A document’s keywords provide high-level descriptions of the content that summarize the document’s central themes, concepts, ideas, or arguments. These descriptive phrases make it easier for algorithms to find relevant information quickly and efficiently. It plays a vital role in document processing, such as indexing, classification, clustering, and summarization. Traditional keyword extraction approaches rely on statistical distributions of key terms in a document for the most part. According to contemporary technological breakthroughs, contextual information is critical in deciding the semantics of the work at hand. Similarly, context-based features may be beneficial in the job of keyword extraction. For example, simply indicating the previous or next word of the phrase of interest might be used to describe the context of a phrase. This research presents several experiments to validate that context-based key extraction is significant compared to traditional methods. Additionally, the KeyBERT proposed methodology also results in improved results. The proposed work relies on identifying a group of important words or phrases from the document’s content that can reflect the authors’ main ideas, concepts, or arguments. It also uses contextual word embedding to extract keywords. Finally, the findings are compared to those obtained using older approaches such as Text Rank, Rake, Gensim, Yake, and TF-IDF. The Journals of Universal Computer (JUCS) dataset was employed in our research. Only data from abstracts were used to produce keywords for the research article, and the KeyBERT model outperformed traditional approaches in producing similar keywords to the authors’ provided keywords. The average similarity of our approach with author-assigned keywords is 51%.

## Introduction

As the amount of unstructured textual data increases exponentially, the need for automatically processing and extracting knowledge from it becomes increasingly important. An essential step towards automatic text processing is the automatic indexing of unstructured text, tagging the text with a predefined vocabulary, taxonomy, thesaurus, or ontology, enabling the algorithms to find relevant information more quickly and effectively (Khan et al., 2019). A practical approach for automatic text indexing is to summarize the main themes, concepts, ideas, or arguments into a small number of phrases or words, called key phrases or keywords, which are meaningful words or phrases that describe the document at a highly abstract level. However, most documents do not contain author-provided keywords or key phrases, and manually identifying key phrases for extensive document collections is labor-intensive. Keyword extraction is a technique to automatically extract a word or subset of words from a document. Extracting a set of components containing one or more words from a single document is essential in many applications, *e.g.*, keywords are often used to query the system, extract the summary of a document, automatic indexing, automatic integration, document management, high-level semantic description text, the document or grouping of web sites, cross-category retrieval, construction of domain-specific dictionaries, named entity recognition and topic recognition, and so on.

A keyword or key phrase is a word that precisely and accurately describes the content of a document, in whole or in part (Tahir et al., 2021). A keyword is a unigram, while a key phrase is an N-gram, *i.e.,* more than one word. For example, “family” is a keyword, and ”family members” is a key phrase. The reason people prefer the key phrase over the keyword is because the key phrase contains more information and meanings. It is contextually related to the keyword, and its contextual meaning can vary depending on the text and environment. For example, the word “bank” could mean an organization, or it could be the bank of a river. Therefore, context is essential to understanding the real meaning of a word. The context of a word reflects its meaning. Context-based features are pretty helpful for extracting keywords, and this process is intrinsic to extracting the central meaning of a text document and expressing the issues discussed in it. In general, even if we do not know a term, we can guess its meaning based on the context in which the term is used. For example, simply indicating the previous or next word of the phrase of interest might be used to describe the context of a phrase (Wang & Li, 2017). The proposed work relies on identifying a group of important words or phrases from the document’s content that can reflect the authors’ main ideas, concepts, or arguments. Thus, keywords or key phrases must be contextually and semantically meaningful accepted terms containing one or more unigram words.

Existing keyword extraction techniques rely on distributional features that cannot understand the context and meaning of sentences and documents. Distributional characteristics do not help the keyword extraction algorithm to understand the context and semantics of sentences and documents as most of the features only represent statistical distributions of the sentences. Moreover, the use of semantic knowledge provided by the public knowledge base to analyze the semantic relationships of phrases has limitations. Firstly, most knowledge bases have a limited vocabulary that does not adequately cover rapidly growing technical terms. Secondly, they provide only general meanings of common words, which contributes very little to the analysis of domain-specific corpora. For example, the word “neuron” commonly refers to the basic components of the brain and spinal cord of the central nervous system, so it is widely associated with the neocortex or sensorimotor. In contrast, in the domain of machine learning, a “neuron” usually refers to a node in a neural network that performs linear or nonlinear computation. Some closely related words to “neuron” may be sigmoid or backpropagation in the machine learning domain.

The extraction of keywords is extremely demanding, and manual extraction is time-consuming; for scientific papers published on a single day, it is almost impossible to extract keywords due to their volume manually. To quickly utilize keywords, we proposed a method that pulls keywords from scientific documents to analyze the effects of context word embedding. Though it is easy to extract keywords and key phrases from the corpus of long sentences, it is somewhat difficult to extract the same from a shorter sentence. The contextual word embedding model will effectively extract keywords and key phrases semantically and contextually in our proposed approach. The suggested one is extracting keywords, focusing on contextual features, and outperforming some traditional methods.

We examined a variety of recent keyword extraction approaches. For extracting keywords from documents, a variety of methods and methodologies have been developed. Term frequency, POS tagging, support vector machine, conditional random fields, and other techniques have all demonstrated improved results in extracting keywords, but to enhance the link between words and sentences, they must be properly captured. We believe that by employing the contextual word embedding approach we may obtain better outcomes in extracting keywords from texts. Our contextual word embedding technique extracts keywords/key phrases from scientific publications automatically in this research. Because each word is distinct depending on the context, the attention is on the contextual aspects of the words. We employed a KeyBert model for contextual word embedding instead of a frequency-based technique to encode a text in a vector. The KeyBert model captures a term’s semantics as well as the context in which it appears in a text. Traditional approaches are not effective when it comes to synonyms. To locate synonyms for the extracted phrases, we utilized WORDNET. Furthermore, the suggested task’s performance is compared to that of other established methodologies.

## Literature Review

The methods for finding keywords and key phrases can be divided into the following categories: statistical methods, clustering-based methods, graph-based methods, embedding-based methods, and machine learning methods.

### Statistical-based methods

Statistical-based methods use deterministic mathematical functions to identify phrases with unusual frequencies. Different algorithms interpret the term “unusual” in different ways. For example, TF-IDF (Sparck Jones, 1972) identifies a word with a high frequency in a few specific documents rather than being evenly distributed over the corpus. Similarly, Likely (Paukkeri et al., 2008) selects phrases by taking the ratio of a rank value of a phrase in the documents to its rank value in the referenced corpus. The rank is calculated as the relative N-gram frequency of the phrase. RAKE (Rose et al., 2010) identifies unusually frequent phrases by analyzing how often it coexists with other phrases. It scores a phrase as the ratio of co-occurrence frequency with other phrases and their frequency. Wartena, Brussee & Slakhorst (2010) compare the co-occurrence distributions of phrases in a document with their frequency distributions in the whole corpus. KP-Miner (El-Beltagy & Rafea, 2009) is a key phrase extraction system that utilizes several kinds of statistical information far away from the TF-IDF score. It follows an impressive process of candidate phrase filtering and uses a scoring feature like TF-IDF. At the same time, external resource-based co-occurrence statistics and statistical measurement began to be used to calculate semantic similarities among documents candidate terms. Statistical-based approaches usually do not require any additional resources apart from the raw data statistics of phrases from the corpus and documents. This allows statistical-based approaches to be easily reimplemented. However, they can be frequency-sensitive, favoring high-frequency phrases, thus preventing the algorithms from identifying keyphrases having low frequencies.

### Clustering-based methods

Clustering-based approaches apply clustering algorithms to group candidate phrases into topic clusters, then the most representative ones from each cluster are selected as key phrase. Liu et al. (2009) apply hierarchical, spectral, and affinity propagation clustering algorithms that group semantically related phrases using Wikipedia and co-occurrence frequencies. Key phrases are the phrases close to the centroid of each cluster. Bracewell, Ren & Kuriowa (2005) cluster phrases by arranging all unigram words to their clusters, then multi-gram phrases are assigned to clusters containing the component unigrams. If no unigram cluster is found, candidates are assigned to their clusters. Finally, centroids from the top-k scored clusters are extracted as key phrases. Pasquier (2010) proposes inducing topic distributions from groups of semantically related sentences using both cluster algorithms and LDA. Key phrases are scored considering distributions of topics over clusters, the distributions of phrases over topics, and the size of each cluster. KeyCluster (Gagliardi & Artese, 2020) attempts to extract key phrases that address all the important topics in a document. Extracting keyphrases from each topic cluster treats that topics are equally important in an article. In reality, however, there exist minor topics that are unimportant to an article. Hence, they should not have keyphrases representing them.

### Graph-based methods

Graph-based approaches score phrases using graph ranking algorithms by representing a document as a graph. Each phrase corresponds to a vertex. Two vertices are connected if pre-identified relations, such as phrase co-occurrence relations, are found in predefined ones window size. HITS (Kleinberg, 1999) and PageRank (Brin & Page, 1998) recursively compute the importance of a vertex in a graph by analyzing both the number of neighboring vertices it connects to and the importance of each neighbor vertex. Applying link analysis algorithms to keyphrase extraction assumes that (1) an important phrase should have high frequency, such that it co-occurs more often with other phrases, and (2) a phrase selectively co-occurs with one or a few highly frequent ones can also be important. Mihalcea and Tarau introduced TextRank (Mihalcea & Tarau, 2004), which applies PageRank to automatic keyword extraction (AKE) by representing documents as undirected and unweighted graphs, considering only frequency and co-occurrence frequency features. Wan & Xiao (2008) propose to use phrase co-occurrence frequencies as weights collected from both the target document and its k nearest neighbor documents identified using document cosine similarity measure. The approach essentially expands a single document to a small one by adding a few topic-wise similar documents to capture more statistical information. Wang, Liu & Wang (2007) use synset in WordNet to obtain the semantic relations for a pair of phrases.

### Embeddings-based methods

Words are represented numerically in a way that is readily to process by computer algorithms. The representative techniques based on the co-occurrence matrix are Latent Semantic Analysis (LSA) (Deerwester et al., 1990) and Latent Dirichlet Allocation (LDA) (Blei, Ng & Jordan, 2003). Mikolov et al. (2013) proposed word embedding, but they also introduced the well-known Continuous Bag-of-Words (CBOW) and Skip-gram continuous models. In addition, sentence embeddings (Doc2Vec Lau & Baldwin, 2016 or Sent2vec Pagliardini, Gupta & Jaggi, 2017) and the famous GloVe (Global Vectors) (Pennington, Socher & Manning, 2014) approach are exploited by keyphrase or keyword extraction approach. EmbedRank (Bennani-Smires et al., 2018) retrieves candidate phrases based on POS. EmbedRank represents candidate and document sentences in high-dimensional sets vector form using sentence embeddings (Sent2vec or Doc2Vec). At last, the cosine similarity of the candidate phrase embedding with the document embedding is used by the algorithm to provide a ranking to the candidate phrases. The Reference Vector Algorithm (RVA) was introduced by Papagiannopoulou & Tsoumakas (2018), a keyword extraction strategy whose fundamental concept is to exploit the word embeddings (GloVe vectors) trained on the content under review.

### Machine learning

Based on the learning algorithms employed, supervised machine learning approaches treat AKE as either a classification or a ranked learning problem. When treating AKE as a binary classification problem, the goal is to train a classifier that decides whether a candidate can be a key phrase. KEA (Witten et al., 1999) is one of the first keywords or keyphrase extraction methods that calculate each candidate phrase’s first occurrence and TF-IDF score, *i.e.,* the position of the first occurrence of the sentence and the utilizing of Naive Bayes as a learning algorithm. Hulth (2003) proposes a strategy based on language knowledge. In particular, four features are computed for the every candidate sentence in the training set. The frequency within the document, the frequency of collection, the initial occurrence part-of-speech (POS) sequence of tag and associated position. The control induction technique with bagging in this case is the machine learning methodology.

Jo & Lee (2015) use a Deep Belief Network (DBN) that connects to a logistic regression layer to learn a classifier. The model does not require any manually selected features. Instead, it uses a greedy layer-wise unsupervised learning approach (Hinton, Osindero & Teh, 2006) to understand the features of one layer once a time automatically. Zhang et al. (2016) propose a deep learning model using Recurrent Neural Network (RNN), combining keywords and context information to extract key phrases. The network has two hidden layers, where the first one aims to capture the keyword information, and the second layer extracts the key phrases based on the encoded keywords information from the first layer. Li, Zhu & Lu (2015) use word embeddings to represent words, then apply Euclidean distances to identify the top-N-closest keywords as the extracted keywords. Meng et al. (2017) (seq2seq) introduced a productive model for predicting key phrases utilizing an encoder–decoder framework that can capture the text’s semantic meaning through a deep learning approach.

At last, Basaldella et al. (2018) suggested a Bi-LSTM RNN that may exploit the previous and next context of a word. To tokenize words, the text material is first broken down into sentences, and each word is given its own word embedding. Finally, a Bi-LSTM RNN is fed the word embeddings. In this sense, Alzaidy, Caragea & Lee Giles (2019) proposed an approach that combines a Bi-LSTM layer to model the sequenced text information with a Conditional Random Field (CRF) layer to model output dependencies.

## Proposed Methodology

**Figure 1:**
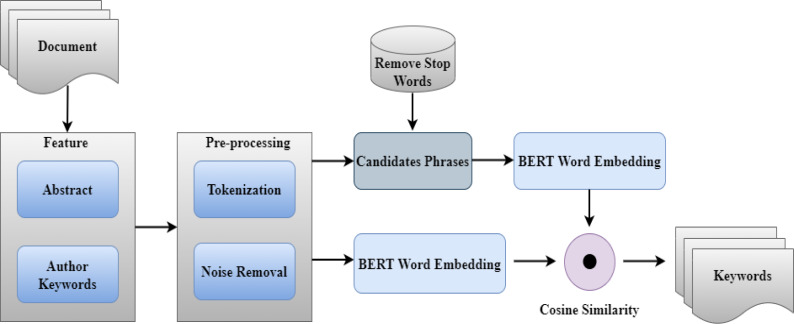
Flow diagram of the proposed approach.

Our method involves extracting keywords on the basis of their contextual relationship to the phrase. In contrast to statistical methods based on frequency, when extracting keywords, they only consider frequency and co-occurrence of terms, so we focus on the relevance between words in the context of the sentence. As a result, word and phrase semantic and contextual information is utilized in the extraction process. The contextual attributes of words in sentences are extracted by applying context word embedding using BERT (Bidirectional Encoder Representations from Transformers) (Devlin et al., 2018). For example, the context word embedding models (Roman et al., 2021; Aljuaid et al., 2021) suggest a word in context or a context-dependent word vector. The BERT has shown promising results in other areas such as Sentiment classification (Aljuaid et al., 2021) and Named Entity Recognition (NER) (Gao et al., 2019; Haider et al., 2021). KeyBERT is a keyword extraction method that uses BERT embeddings to extract keywords that are the most representative of the underlying text document. It is an unsupervised method of extracting keywords from a text. KeyBERT consists of three consecutive steps, such as Candidate Keywords or Keyphrases, BERT Embedding, and Similarity. Our approach is shown in Fig. 1.

### Data collection

We used the Journal of Universal Computer Science (JUCS) (Haider et al., 2021) data as a source. The detailed statistical data of the dataset are shown in Table 1. To evaluate our approach, we have carefully selected a dataset that contains research articles of the Computer Science domain. The dataset comprises scientific documents from the Journal of Universal Computer Science. The reason behind the selection of the JUCS dataset is that it contains papers from multiple areas of the Computer Science domain, and the Author’s provided keywords which plays a significant role in a comprehensive evaluation. Furthermore, the JUCS datasets contain the metadata of the research articles. We extracted the research articles’ metadata from these datasets and selected metadata like abstract and keywords. The following is the reason behind choosing specific data as a feature:

**Table 1:** Dataset statistics.

Features	Records
Total number of research papers	1460
Total number of classes (categories) at root level	13
Total number of journals or conferences or workshops	01
Total number of research papers without keywords’ section	31

• The abstract of the paper contains prospective terms that might be used in identifying the keywords of a research article.
• Keywords are assigned by the authors of the papers themselves and are usually from relevant fields.

We took 1,363 research articles for keyword extraction. The dataset of scientific documents contains special characters; for that reason, we use standard regular expressions and available toolkits for data cleaning and preprocessing.

### Candidate keywords or keyphrases

**Figure 2:**
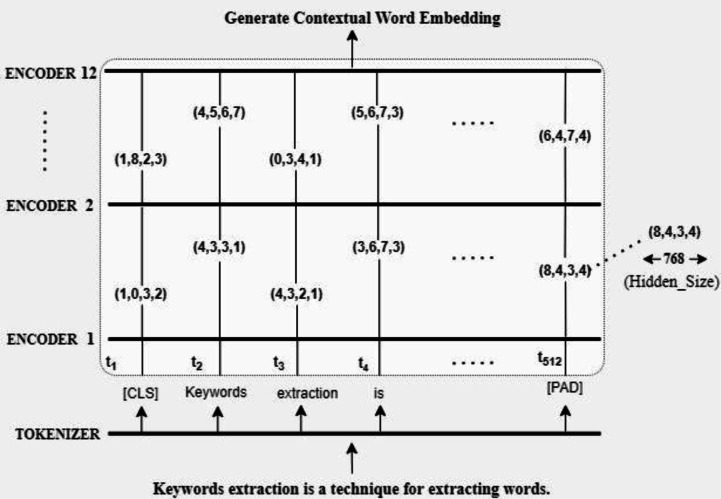
Contextual word embedding by BERT.

Firstly, we start by extracting n-grams *i.e.,* candidate keywords or key phrases from a document’s abstract for extracting keywords. N-grams are nothing more than a string of n-consecutive tokens. We utilize Scikit-Learns Count Vectorizer to get a list of candidate n-grams. Count Vectorizer ranks the n-grams based on their frequency in the original document. Count Vectorizer to keep things simple instead of focusing on noun phrases. We may now select the keyword length and transform them into key phrases. It’s also a simple method to get rid of stop words. To alter the size of the resultant candidates, used the n-gram-range function. For example, if we change it to (3, 3), the candidates will be sentences that include three keywords. The variable candidates are simply a collection of strings that contain our candidate keywords. The flow of extracting candidate keywords or key phrases is shown in Fig. 2.

### Embeddings

**Figure 3:**
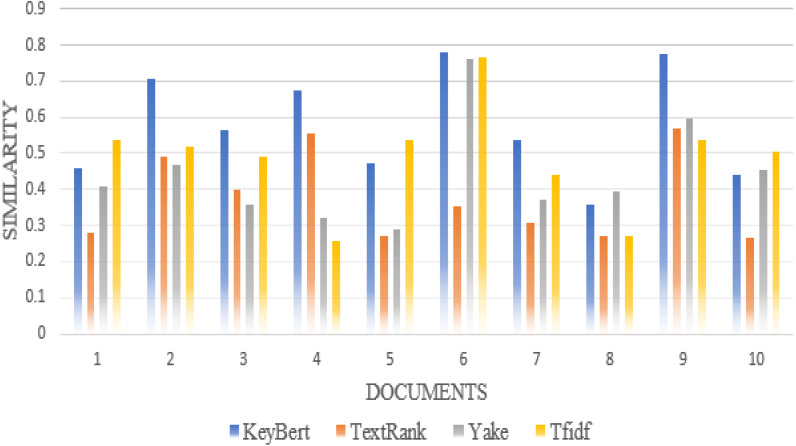
Comparison of each document similarity with author-assigned keywords in different approaches.

Next, we transform both the abstract of the scientific document and the n-gram *i.e.,* keywords or key phrases into numeric data. For this purpose, we employ the BERT model, which has demonstrated good performance in both similarity and paraphrasing tasks. BERT architecture is shown in Fig. 3. BERT demands data to be in a specified format, with special tokens to indicate the start ([CLS]) and end ([CLS]) of sentences ([SEP]). In addition, we must tokenize our text into tokens that match BERT’s vocabulary. BERT requires input ids, a series of numbers that link each input token to its index number in the BERT tokenizer vocabulary, for each tokenized phrase. For BERT this will be a vector with 768 elements. We may utilize those 768 values as contextual word embeddings because they contain our numerical representation of a single token. We are dealing with a tensor of size 768 by the number of tokens. Each token produced by the encoders is represented by one of these vectors. We can use these tensors to generate semantic representations of the input sequence by transforming them.

### Similarity measure

**Figure 4:**
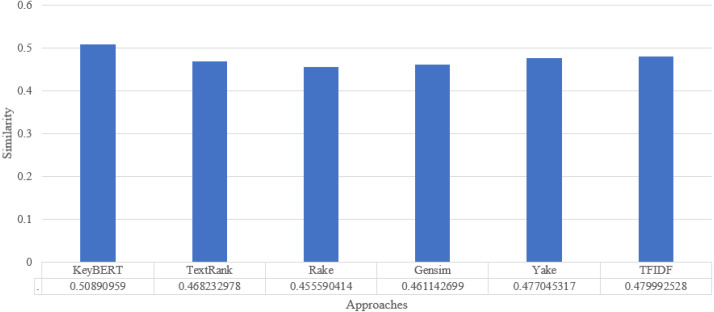
Keyword extraction analysis with different approaches.

In the final phase, we looked for n-gram *i.e.,* candidates comparable to the document. The choices that are the most similar to the document are more likely to be suitable keywords/keyphrases expressing the document. The greater the similarity, the more relevant and representative the keyword is to the source document. The relevancy of candidates keywords and document is shown in Fig. 4. Cosine similarity is used to determine the similarity between the candidates and the document abstract because it works well at large dimensionality. The resultant keywords are the five most comparable choices to the input document.

(1) \begin{eqnarray*}Similarity=COS(W.S).\end{eqnarray*}

The similarity is defined as the cosine similarity among a word’s word embedding vector “W” and the sentence embedding vector “S”, as shown in Eq. (1).

## Experimental Result

In the experimental results, we discuss the experimental results of our approach with the keyword assigned by the author. The closer the extracted keywords are to the keyword assigned by the author, the better the performance.

**Figure 5:**
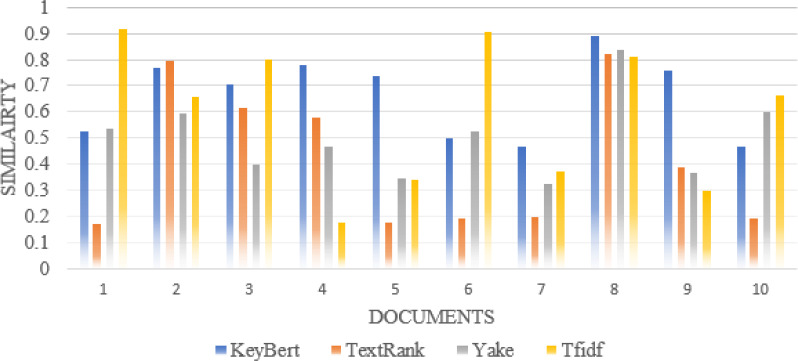
Comparison of each document similarity with author-assigned keywords in different approaches using Wordnet synonyms.

Furthermore, we compare the similarity ratio of the extracted keywords of each scientific document with our approach and the other traditional approaches such as TextRank, Rake, Gensim, Yake (Labusch et al., 2019), TF-IDF. Thus, we can see in Fig. 5 that the performance of our approaches is better than the traditional approaches in most individual scientific documents.

**Figure 6:**
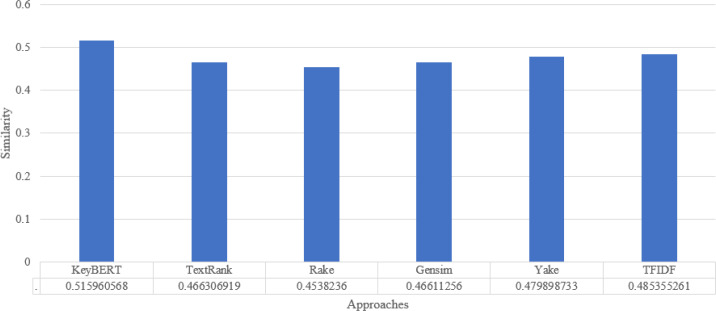
Keyword extraction analysis with different approaches using wordnet synonym.

To analyze the overall performance of our approach, we compute the average similarity ratio of all keywords extracted from the abstracts of the scientific documents of our approach and the other existing approaches. As we can see in Fig. 6, the average similarity ratio of our approach is better than that of other traditional approaches such as Text Rank, Rake, Gensim, Yake, TF-IDF.

In the second experiment, we take the synonyms of the keywords assigned by the Author and the keywords extracted from scientific documents of existing automatic approaches and our approach because the traditional or existing approach extracts keywords based on the frequency and co-occurrence of words and has no idea about the word’s synonyms.

**Figure 7:**
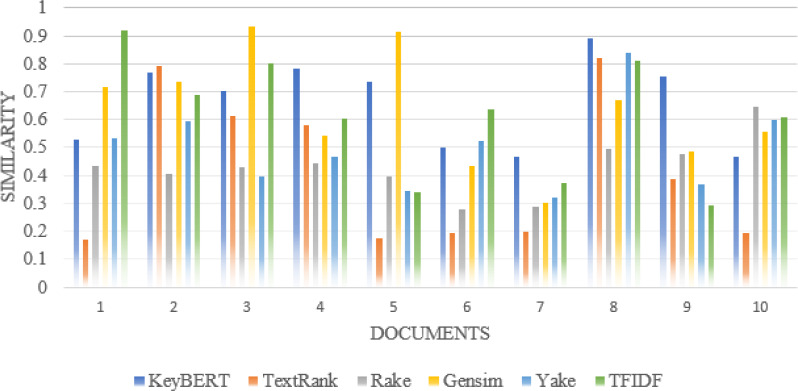
Comparison of each document similarity with Author assigned keywords in different approaches using Wordnet synonyms.

**Figure 8:**
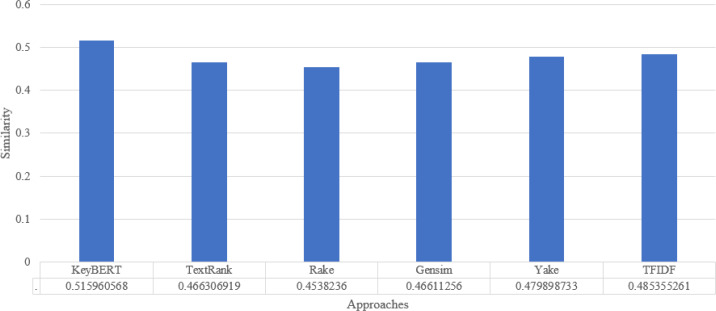
Keyword extraction analysis with different approaches using Wordnet synonym.

For synonyms, we use Wordnet. WordNet is a useful lexical resource. Its unique semantic network helps discover word relationships, synonyms, grammar, and other topics. This supports NLP tasks such as sentiment analysis, automatic language translation, and text similarity, among others. We apply all the procedures we used in Experiment 1 to test the performance of keyword extraction with wordnet synonyms. Thus, we can see in Fig. 7 that the performance of our approach is better than the traditional approaches in most individual scientific documents. Figure 8 shows the average similarity ratio of our approach using wordnet synonyms is better than traditional approaches.

To evaluate the percentage gain that our approach achieves compared to traditional approaches, we use the percentage increase formula is shown in Eq. (2).

(2) \begin{eqnarray*}\text{%}~increase= \frac{\mathrm{X}2-\mathrm{X}1}{\mathrm{X}1} \end{eqnarray*}

where,

• X1 = Initial Value
• X2 = Final Value

KeyBERT V_1_ = Represent first experiment that we extract keywords with different approaches and find the similarity with Author assigned keywords.

KeyBERT V_2_ = Represents the second experiment where we take synonyms of the extracted keywords using Wordnet and compare them with other approaches.

As shown in Table 2, we obtain a better percentage gain than other approaches. In Table 2, the first row shows the percentage gain of KeyBERT V1 compared to other methods. As can be seen, KeyBERT v1 achieves 9%, 12%, 10%, 7% and 6% gain compared to TextRank, Rake, Gensim, Yake and TF-IDF, respectively. KeyBERT v2 thus achieves 11%, 14%, 11%, 8% and 6% compared to TextRank, Rake, Gensim, Yake and TF-IDF, respectively.

**Table 2:** KeyBert percentage gain as compared to Traditional methods.

Approaches	TextRank	Yake	TF-IDF
KeyBert V_1_	9%	7%	6%
KeyBert V_2_	11%	8%	6%

## Conclusion

In this research, we proposed extracting keywords *via* contextual word embedding. Data from the Journals of Universal Computer Systems (JUCS) was used for validation. The proposed method outperforms previous methods by extracting keywords and focusing on contextual information. The results showed that the keywords retrieved with KeyBert are remarkably like the keywords supplied by the author. Based on the empirical findings, we propose that keyword extraction algorithms consider the semantics and context of the text, which statistical methods currently ignore. In this study, keywords were initially extracted from the article’s abstract using KeyBert. Then, keywords were extracted using standard techniques such as TF-IDF, Yake, Rake, Gensim and TextRank for comparison purposes. Afterward, cosine similarity was computed with author-assigned keywords. Similarly, in the second experiment, we enhanced the extracted author-assigned keywords with the help of Wordnet; then, the cosine similarity was computed. From the results, it is evident that results of context-based keyword extraction are better than traditional approaches.

In future this study can be expanded by looking at other sections of research paper such as introduction and conclusion to uncover more closely related to the authors’ keywords. Since the keywords given by authors are mostly from these sections, we will use Deep Learning model with Bert to improve the similarity of approach with author-assigned keywords.

##  Supplemental Information

10.7717/peerj-cs.967/supp-1 Supplemental Information 1 Code used in the current study

10.7717/peerj-cs.967/supp-2 Supplemental Information 2 Data Set used in the current study

10.7717/peerj-cs.967/supp-3 Supplemental Information 3 DataSet Source

## Additional Information and Declarations

### Data Availability

The following information was supplied regarding data availability: The datasets and code used is are available in the Supplementary Files. The data set source is: Roman M, Shahid A, Uddin MI, Hua Q, Maqsood S. 2021. Exploiting contextual word embedding of authorship and title of articles for discovering citation intent classification. Complexity 2021:5554874 https://doi.org/10.1155/2021/5554874.

## References

[ref-1] Aljuaid H, Iftikhar R, Ahmad S, Asif M, Afzal MT (2021). Important citation identification using sentiment analysis of in-text citations. Telematics and Informatics.

[ref-2] Alzaidy R, Caragea C, Lee Giles C (2019). Bi-LSTM-CRF sequence labeling for keyphrase extraction from scholarly documents.

[ref-3] Basaldella M, Antolli E, Serra G, Tasso C (2018). Bidirectional lstm recurrent neural network for keyphrase extraction.

[ref-4] Bennani-Smires K, Musat C, Hossmann A, Baeriswyl M, Jaggi M (2018). Simple unsupervised keyphrase extraction using sentence embeddings.

[ref-5] Blei DM, Ng AY, Jordan MI (2003). Latent dirichlet allocation. Journal of Machine Learning Research.

[ref-6] Bracewell DB, Ren F, Kuriowa S (2005). Multilingual single document keyword extraction for information retrieval.

[ref-7] Brin S, Page L (1998). The anatomy of a large-scale hypertextual web search engine. Computer Networks and ISDN Systems.

[ref-8] Deerwester S, Dumais ST, Furnas GW, Landauer TK, Harshman R (1990). Indexing by latent semantic analysis. Journal of the American Society for Information Science.

[ref-9] Devlin J, Chang M-W, Lee K, Toutanova K (2018). Bert: pre-training of deep bidirectional transformers for language understanding.

[ref-10] El-Beltagy SR, Rafea A (2009). KP-Miner: a keyphrase extraction system for English and Arabic documents. Information Systems.

[ref-11] Gagliardi I, Artese M (2020). Semantic unsupervised automatic keyphrases extraction by integrating word embedding with clustering methods. Multimodal Technologies and Interaction.

[ref-12] Gao Z, Feng A, Song X, Wu X (2019). Target-dependent sentiment classification with BERT. IEEE Access.

[ref-13] Haider S, Afzal M, Asif M, Maurer H, Ahmad A, Abuarqoub A (2021). Impact analysis of adverbs for sentiment classification on Twitter product reviews. Concurrency and Computation: Practice and Experience.

[ref-14] Hinton GE, Osindero S, Teh Y-W (2006). A fast learning algorithm for deep belief nets. Neural Computation.

[ref-15] Hulth A (2003). Improved automatic keyword extraction given more linguistic knowledge.

[ref-16] Jo T, Lee JH (2015). Latent keyphrase extraction using deep belief networks. International Journal of Fuzzy Logic and Intelligent Systems.

[ref-17] Khan AM, Shahid A, Afzal MT, Nazar F, Alotaibi FS, Alyoubi KH (2019). SwICS: section-wise in-text citation score. IEEE Access.

[ref-18] Kleinberg JM (1999). Authoritative sources in a hyperlinked environment. Journal of the ACM (JACM).

[ref-19] Labusch K, Kulturbesitz P, Neudecker C, Zellhöfer D (2019). BERT for named entity recognition in contemporary and historical German.

[ref-20] Lau JH, Baldwin T (2016). An empirical evaluation of doc2vec with practical insights into document embedding generation.

[ref-21] Li Q, Zhu W, Lu Z (2015). Predicting abstract keywords by word vectors.

[ref-22] Liu Z, Li P, Zheng Y, Sun M (2009). Clustering to find exemplar terms for keyphrase extraction.

[ref-23] Meng R, Zhao S, Han S, He D, Brusilovsky P, Chi Y (2017). Deep key phrase generation.

[ref-24] Mihalcea R, Tarau P (2004). Textrank: bringing order into text.

[ref-25] Mikolov T, Chen K, Corrado G, Dean J (2013). Efficient estimation of word representations in vector space.

[ref-26] Pagliardini M, Gupta P, Jaggi M (2017). Unsupervised learning of sentence embeddings using compositional n-gram features.

[ref-27] Papagiannopoulou E, Tsoumakas G (2018). Local word vectors guiding keyphrase extraction. Information Processing & Management.

[ref-28] Pasquier C (2010). Single document keyphrase extraction using sentence clustering and latent dirichlet allocation.

[ref-29] Paukkeri M-S, Nieminen IT, Pöllä M, Honkela T (2008). A language-independent approach to keyphrase extraction and evaluation.

[ref-30] Pennington J, Socher R, Manning CD (2014). Glove: global vectors for word representation.

[ref-31] Roman M, Shahid A, Uddin M, Hua Q, Maqsood S (2021). Exploiting contextual word embedding of authorship and title of articles for discovering citation intent classification. Complexity.

[ref-32] Rose S, Engel D, Cramer N, Cowley W (2010). Automatic keyword extraction from individual documents. Text Mining: Applications and Theory.

[ref-33] Sparck Jones K (1972). A statistical interpretation of term specificity and its application in retrieval. Journal of Documentation.

[ref-34] Tahir N, Asif M, Ahmad S, Malik M, Aljuaid H, Butt M, Rehman M (2021). FNG-IE: an improved graph-based method for keyword extraction from scholarly big-data. PeerJ Computer Science.

[ref-35] Wan X, Xiao J (2008). Single document keyphrase extraction using neighborhood knowledge.

[ref-36] Wang J, Liu J, Wang C (2007). Keyword extraction based on pagerank.

[ref-37] Wang L, Li S (2017). PKU_ICL at SemEval-2017 task 10: Keyphrase extraction with model ensemble and external knowledge.

[ref-38] Wartena C, Brussee R, Slakhorst W (2010). Keyword extraction using word co-occurrence.

[ref-39] Witten I, Paynter G, Frank E, Gutwin C (1999). C. nevillmanning, kea: practical automatic keyphrase extraction.

[ref-40] Zhang Q, Wang Y, Gong Y, Huang X-J (2016). Keyphrase extraction using deep recurrent neural networks on twitter.

