# Supplementary material for: Impact analysis of keyword extraction using contextual word embedding
Source: PeerJ Comput Sci. 2022 May 30;8:e967. doi: 10.7717/peerj-cs.967 (PMC9202614; doi:10.7717/peerj-cs.967)
Supplement: Supplemental Information 3 [file peerj-cs-08-967-s003.docx]

Please note that the data set is taken from an open source journal, Journal of Universal Computer Science. Hence it is publicly and freely available at https://www.jucs.org/. This has also been mentioned i the manuscript section 3.1 line 1 , cited at reference 35
